# Mild strain cross protection of tristeza: a review of research to protect against decline on sour orange in Florida

**DOI:** 10.3389/fmicb.2013.00259

**Published:** 2013-09-06

**Authors:** Richard F. Lee, Manjunath L. Keremane

**Affiliations:** National Clonal Germplasm Repository for Citrus and Dates, Agricultural Research Service, United States Department of AgricultureRiverside, CA, USA

**Keywords:** biological indexing, strain differentiation, serology, stem pitting, mild isolate selection

## Abstract

Tristeza, caused by *Citrus tristeza virus* (CTV), has long been present in Florida but outbreaks of decline on sour orange rootstock were occasional events until the late 1970s. Sour orange rootstock was valued for the high quality of fruit produced and was widely used because of its tolerance of citrus blight, a disease of unknown etiology. Research was directed towards the selection and screening of mild strains of CTV which could protect against sour orange decline strains. Following the introduction of *Toxoptera citricida* (also known as the brown citrus aphid) in 1995 there was a greater concern for maintaining production of existing blocks of citrus on sour orange rootstock. Availability of the CTV genome sequence around the same time as well as molecular characterization of *in planta* CTV populations led to the selection of mild CTV isolates which when inoculated into existing field trees, extended the productive life of the groves and enabled a more graduate replanting of trees on CTV-tolerant rootstocks. The history of CTV in Florida and the methods developed to select mild isolates for use for mild strain cross protection will be reviewed.

## TERMINOLOGY

For purpose of this review, we refer to strains of CTV as causing a specific biological activity consistently; e.g., mild strains of CTV will always produce mild symptoms even on susceptible hosts, decline strains will consistently cause decline on sour orange rootstock. The term isolate is used to describe the viral population of CTV obtained from a field source, and the isolate may be composed of a mixture of strains. Genotypes of CTV are identified by the use of specific methods using molecular markers that are dependent on genome sequence.

## INTRODUCTION

*Citrus tristeza virus* (CTV) is the most important viral disease of citrus worldwide. Since the first outbreaks in South America in the 1940s, CTV has killed more than 85 million trees worldwide (Lee et al., 1992; Bar-Joseph et al., 2010). CTV occurs as strains which cause a variety of biological symptoms on various hosts. Mild strains of CTV (CTV-M) cause no detectable symptoms in common scion/rootstock combinations and produce very slight to no symptoms in Mexican lime indicator plants under ideal conditions. Some CTV strains cause decline of scions on sour orange rootstock (CTV-D); these strains are apparent in areas which use sour orange as rootstock. The decline on sour orange due to CTV-D may be rapid, within weeks, or more gradual taking up to 18–24 months for the trees to gradually decline (Lee and Brlansky, 1990). Some strains of CTV produce stem pitting symptoms in scions (CTV-SP). CTV strains which induce stem pitting on grapefruit (CTV-SPg) are the most commonly seen stem pitting strains, but some strains also induce stem pitting on sweet orange scions (CTV-SPs). Some CTV-SP strains will stem pit only grapefruit and not sweet orange, others stem pit only sweet orange and not grapefruit (Lee and Rocha-Pena, 1992), and others will stem pit both grapefruit and sweet orange. The symptomatology of stem pitting can be varied as well as the effect the stem pitting has on the tree vigor and yield. The most severe stem pitting are the very small pits produced in the bark with corresponding fine pegs in the wood of stems and branches, with a gumming occurring in the pitted areas. Often ropey-like large pits occur on the trunk of trees, especially in grapefruit trees, and while detrimental to tree vigor over time, the economic impact on the tree is usually less than trees showing the small pitting with gum deposits in the pitted area (Bar-Joseph et al., 1989). Another symptom associated with CTV is seedling yellows (CTV-SY) which is expressed by a yellowing on lemon, grapefruit, or sour orange followed by a stunting of growth in these hosts. A standardized host range using Mexican lime, sour orange, Madame vinous sweet orange, and Duncan grapefruit seedlings and a sweet orange budded onto sour orange as standard indicator plants has been developed and used as a method for determining the biological activity of a specific CTV isolate and to enable comparison of biological activities of CTV isolates from various areas of the world (Garnsey et al., 1987, 2005).

Once CTV becomes established in a citrus growing area, what methods are available for control of the disease? If CTV-D is the predominate CTV strain in an area that has mostly trees on sour orange rootstock, the disease may be managed by simply replanting trees on a rootstock tolerant to CTV decline. For CTV-SP strains, options can include implementation of mild strain cross protection, development of CTV resistance in commercially desirable cultivars via genetic engineering methods, and/or development of CTV resistance by conventional plant breeding method using the immunity against CTV that is present in *Poncirus trifoliata*.

Genetic engineering resistance to CTV in commercially desirable varieties may take 15–20 years while the use of conventional plant breeding is a longer term endeavor requiring several decades. Mild strain cross protection may be implemented immediately if protective mild strains have been selected ahead of time, or in less than a decade if no pre-selection of mild strains has been done.

## USE OF MILD STRAIN CROSS PROTECTION IN VARIOUS CITRUS AREAS

Mild strain cross protection is defined as the phenomenon which occurs when a mild isolate of a virus is introduced into a plant and that virus prevents the expression of the symptoms of a severe isolate of the same virus that is later introduced into the same plant (Lee et al., 1987). Lee et al. (1987), based on observations in Florida, suggested that an ideal CTV strain for cross protection should occur in relative high virus concentration in the plant tissue, should express only mild symptoms in all hosts which may be planted in the region, have the ability to quickly move into new growth flushes, and should be easily aphid transmitted so that once established in a tree or area, it would spread to become the predominate strain in other trees in the area. Mild strain cross protection should not be viewed as a permanent cure to protect against the economic losses caused by severe isolates of CTV, and it is not a form of virus resistance. Rather, mild strain cross protection is a means to extend the economic life of citrus (Lee et al., 1992). Management of CTV by mild strain cross protection should be considered only as a last resort where no other management options are available.

Since mild strain of CTV is a relative term, we will define a mild strain of CTV according to Florida standards: a mild strain of CTV is a strain of CTV that produces only very mild or slight vein clearing and stem pitting on Mexican lime, a very sensitive indicator plant, under optimal cool temperature which favors CTV symptom development. Under most climatic conditions, Mexican lime and other sensitive indicator plants for CTV need to be tested by serological assays to verify the presence of CTV. CTV-M isolates selected in Florida have proven to be the mildest upon comparison with other mild strains from other countries based on host range testing conducted at the USDA ARS Exotic Citrus Pathogen Quarantine, Beltsville, MD, USA (Garnsey et al., 1987, 1991).

Mild strain cross protection has been used successfully in many citrus growing areas to continue production of citrus despite the presence of severe isolates of CTV. In Australia, severe stem pitting on grapefruit was a problem beginning in the 1940s (Fraser and Broadbent, 1979). Apparent mild isolates of CTV were collected from surviving trees and evaluated in field trials (Thornton and Stubbs, 1976). Following aphid transmissions, selection of mild isolates from the aphid transmitted isolates and further evaluations, for the past 35 years all commercial grapefruit trees in Australia have been inoculated with mild isolate PB61 (Zhou et al., 2002).

In Brazil, a similar approach was used in the 1960s where CTV isolates from surviving trees were selected to protect against stem pitting of Pera sweet orange. The IAC selection of Pera has been used for more than 30 years with little breakdown of cross protection. More than 80 million Pera trees have been propagated from this source since its selection in the 1960s (Müller and Costa, 1987; Müller and Rezende, 2004). Two isolates selected in the 1960s for protection of Galego lime against CTV stem pitting and decline have performed well, with protected trees yielding up to five times that of the unprotected trees (Müller and Costa, 1972).

In Peru, Satsuma mandarin budwood was imported from Japan in the 1950s, and this importation is thought to be the source of the severe stem pitting strains of CTV that are present in Peru at the present time (Roistacher, 1988). Screening was performed in a nursery setting where budwood from CTV affected trees was propagated at a single location, and selections were made for trees which grew well despite the presence of severe CTV (Bederski et al., 2005; Roistacher et al., 2010). Additionally, mild attenuated strains of CTV derived by passage through *Passiflora* species, were imported from California (Roistacher and Bar-Joseph, 1987). Using budwood sources infected with the protective strains of CTV, the Navel orange and lime production has increased in the coastal production area of Peru (Bederski et al., 2005; Roistacher et al., 2010).

In South Africa, stem pitting on grapefruit was discovered in the 1940s and presented a production problem (Oberholzer et al., 1949). Selections of CTV were made from surviving grapefruit trees, and these were evaluated for protection against stem pitting when the Citrus Improvement Programme was started in the 1970s (von Broembsen and da Graça, 1976). One of the mild isolates selected came from a Marsh grapefruit planted in 1926 but still producing well when the selection was made in the mid-1970s; this isolate was originally referred to as the Nartia isolate but was later named GFMS12, and was used universally beginning in 1984 to protect grapefruit (Kotzé and Marais, 1976; Marais, 1994). A selection made from lime, later named lime mild strain 6 (LMS6), was found to be effective in lime (van Vuuren et al., 1993), and also was used in sweet orange and mandarin propagations to protect against CTV-SP (Luttig et al., 2002). Later trials also indicated that another CTV selection from grapefruit, named GFMS35, was better at protecting Star Ruby grapefruit and other pigmented grapefruit varieties than the GSMS12 (Marais and Breytenbach, 1996; da Graça, 2010).

In Japan, Hassaku dwarf disease, caused by CTV, severely affects production on Hassaku, *Citrus hassaku*, causing dwarfing and severe stem pitting. An apparently healthy Hassaku, which was later found to be infected with a mild isolate of CTV and citrus vein enation virus, was used as a budwood source (Sasaki, 1979). Trees propagated from this source have grown well, although about 20% of the protected trees showed stem pitting symptoms after 16 years. Other mild isolates of CTV have been identified that protect against stem pitting in Navel orange (Ieki et al., 1997).

## HISTORY OF CTV IN FLORIDA

The first confirmation of CTV occurring in Florida was by Grant (1952); CTV was reported in Orange, Lake, and Highland counties and confirmed by indexing on Mexican lime. Cohen and Knorr (1953) reported the presence of CTV in 27 counties of Florida. At that time, there was no substantial occurrence of decline on sour orange rootstock. It is probable that severe decline strains were prevented from becoming widespread because the predominate rootstock in use in the citrus industry in the early days was sour orange, and a CTV-D isolate would probably have killed the tree, or it would perform so poorly that it would not be propagated. Several nurserymen indicated that some introductions made before the Florida Budwood Registration Bureau existed would not grow on sour orange rootstock (Lee et al., 1997). Following the first confirmed report of CTV in Florida in 1952, there were occasional outbreaks of decline on sour orange rootstock; an outbreak was reported in the Ft. Pierce area in 1956 and other reports of outbreaks in Orange and Polk counties in the 1960s (Norman et al., 1961; Brlansky et al., 1986) A severe outbreak of CTV decline on sour orange was reported in western Orange and southern Lake counties in 1974 (Garnsey and Jackson, 1975). In a survey of registered budwood source trees being used for propagation on sour orange in 1979, 87% of the sweet orange source trees tested positive for CTV and 9% of the grapefruit sources (Garnsey et al., 1980). In the 1980s, CTV decline on sour orange was becoming widespread in the Central and South Ridge growing areas, and in the Indian River on the East Coast and the Flatwoods in Southwest Florida (Brlansky et al., 1986).

The Florida Budwood Registration Bureau (FBRB) was started as a volunteer program in 1953 (Rucks, 1994; Kesinger, 2003). When the Bureau began, registered scion trees had to be tested and found free from CTV and the program allowed trees held by nurseries to be registered as budwood source trees. However, CTV was being naturally spread by aphids within Florida. By 1964, immediately following a severe freeze in December 1962 which increased the demand for budwood, there was concern that if registered scion trees continued to be removed from registered status due to presence of CTV, there would be a severe shortage of budwood. Beginning in 1964, trees were no longer removed from status as budwood sources trees because of CTV (Rucks, 1994); CTV had not been a severe problem at this time in Florida. While budwood source trees infected with CTV could be used for propagations, trees in the FBRB foundation planting at Dundee, FL, USA, were removed when they became CTV infected. This policy of removing CTV infected trees in the Dundee foundation planting was abandoned in 1968 because too many trees were being removed. In 1967–1968, the FBRB established a foundation planting at the Ona Range Cattle Station in Hardee County. The Ona foundation planting was about 1 km away from the nearest commercial citrus planting. By 1972 the Ona foundation planting was no longer used because of the spread of CTV through the planting. In 1989 a 20 acre foundation planting was established at the University of Florida’s Immokalee Research and Education Center, Immokalee. The foundation planting at Immokalee had 28 different registered selections replicated on 22 different rootstocks. CTV began spreading through the foundation planting and in 1992 the foundation trees were inoculated with three different mild isolates of CTV (T30, T26, and T55) so that the industry would be provided with cross protective mild strains. By 1996, the CTV incidence in the Immokalee foundation planting was 37.5% as determined by MCA-13 ELISA to selectively detect severe strains of CTV. Budeyes were no longer cut from the field planting, and subsequently budeyes cut at Immokalee were from a screenhouse. More than 1.1 million budeyes were cut from the Immokalee foundation planting from 1992 to 1998. Registered budwood source trees held by nurseries were located in the field and used to cut budeyes up until the mandatory budwood certification program began in January 1, 1997 (Kesinger, 2003). 

## IMPACT OF CTV ON THE ROOTSTOCK USAGE IN FLORIDA

**FIGURE 1:**
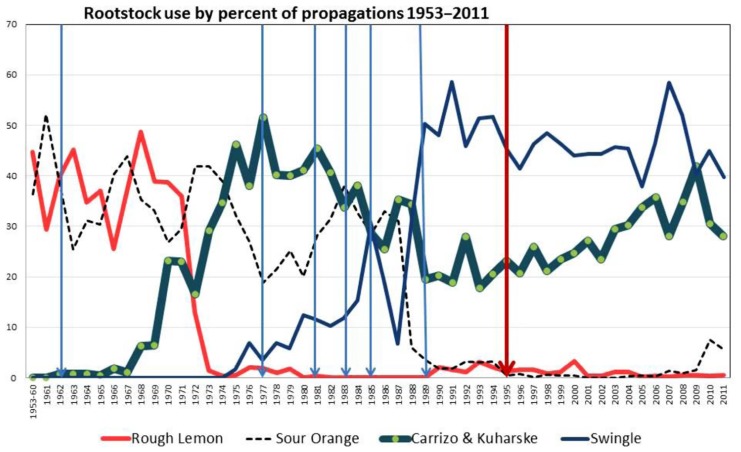
**Rootstock usage in Florida expressed as a percent of the total propagations since the beginning of the Florida Budwood Registration Bureau in 1953.** Major freeze occurrences are indicated by blue vertical lines. In 1995, the program became mandatory (red line) while from 1953 to 1994 the program was voluntary. Data summarized from Kesinger (2011).

**Figure 1** shows the rootstock usage in Florida by percent of propagations since the inception of the Florida program and also the dates of historic freezes as freeze occurrences increase the demand of propagated trees to replace trees lost (Kesinger, 2011). Historically, sour orange was the prevalent rootstock when citrus was grown mainly on the ridge area in Central Florida and the Indian River production area. When new groves were brought into production in the Flatwoods production areas in the 1960s and 1970s, rough lemon was the preferred rootstock. However, a disease of unknown etiology, called citrus blight, began taking trees on rough lemon rootstock out of production beginning when they were 5–7 years of age (Derrick et al., 1992). By the late 1970s, blight was killing about 15% of the trees on rough lemon rootstock each year, and the epidemic of blight spread from the Flatwoods production areas in Southeast and Southwest Florida to the older citrus production areas. While many trees were propagated on Carrizo, and later on the Kuharske selection of Carrizo rootstock (popular because of its tolerance to burrowing nematode), these trees were still subject to losses due to blight. Sour orange continued to be a popular rootstock through the 1980s and lost favor only because the brown citrus aphid (BrCA), *T. citricida*, became established in Florida following its introduction in 1995 (Halbert et al., 2000). Sour orange displays a field tolerance to blight, and produces high quality fruit (Rocha-Pena et al., 1995). The Indian River production area traditionally used sour orange rootstock, and this rootstock has contributed to building the Indian River’s reputation for high quality grapefruit and citrus fruit for the fresh fruit market. Swingle citrumelo became a popular rootstock when the use of sour orange rootstock decreased, because of Swingle’s field tolerance to citrus blight and tolerance to tristeza decline (**Figure 1**). In recent years following the introduction and establishment of Huanglongbing (HLB) in Florida in 2005 (Halbert, 2005), some growers have reverted to sour orange rootstock, especially for grapefruit. HLB had shortened the productive economic life of trees so much that the growers think they would be better off utilizing the advantages of sour orange to get good tree growth and high quality fruit, and also thinking that since the trees are treated with insecticide so often to protect against psyllid infestations, the BrCA, the aphid vector of CTV, should also be less of a problem.

Severe freezes occurred in Florida in January 1977, 1981, December 1983, January 1985, and December 1989 (**Figure 1**; Kesinger, 2011). Each nurseryman had favorite registered trees which produced vigorous budlings when propagated on sour orange rootstock. Following freeze years when demand for trees to replace freeze losses was high, budwood from favored trees was not enough to meet the demand, so budwood from other registered scion trees was used for propagation (Rucks, 1994; Kesinger, 2011). Some of these propagations did not grow well on sour orange rootstock, but if the propagation was on a CTV tolerant rootstock, the effect of CTV was not apparent. This created an ideal situation to distribute CTV-D throughout the Florida citrus industry and created the circumstance for the epidemic of CTV decline which occurred in the 1980s (Brlansky et al., 1986). In 1984, 655 scion sources that were being propagated on sour orange rootstock were biologically indexed on sour orange liners in cooperation with several nurseries (Yokomi et al., 1992). Ten trees were propagated from each budwood source and healthy, mild CTV and CTV-D inoculated controls were included. The stem diameters were measured, and if the diameter was less than 70% of the healthy controls, the plant was declared stunted. Of the 655 scion trees indexed, 18% were stunted. This indicated that many plants were coming from nurseries already infected with CTV-D strains, and the spread of the CTV-D strains was aided by the aphid vectors once the trees were planted (Yokomi et al., 1992).

The BrCA was found in Florida in 1995, and within 2 years was present in all of Florida’s citrus production area (Halbert et al., 2004). A survey was conducted in Southeast Florida in 1994–1995 prior to the BrCA being found in Florida, and 2 years after the BrCA had been found, these same trees were re-sampled to determine changes in incidence, distribution, and severity of CTV isolates in Florida. Serological and molecular assays were conducted, and selected isolates were biologically indexed on sweet orange and grapefruit. A severe CTV-SP was found near Delray Beach, with the incidence of severe strains increasing more than that of the mild strains. With the use of strain group specific probes (Halbert et al., 2004), some trees were found to be infected with up to five different CTV strains. This was the first report of severe CTV-SP occurring in Florida. In 2002–2003, the presence of CTV-SP, causing mild stem pitting on sweet orange and mandarins, was reported in Central Florida and a subsequent survey indicated this stem pitting strain was spreading (Sieburth and Nolan, 2005).

## METHODS USED TO DIFFERENTIATE STRAINS OF CTV

For selection of mild CTV isolates for use for cross protection and for the evaluation of mild strain cross protection experiments, methods are needed to determine and/or predict the biological activity of the isolates. Several methods have been developed, albeit here we will include only the methods actually used in our research to select and screen for mild protective strains of CTV on a timely basis as reported below.

One of the first methods developed for differentiating mild from CTV-D or CTV-SP strains was by the use of monoclonal antibody MCA-13 which recognizes the severe strains of CTV but not the CTV-M strains (Permar et al., 1990). Pappu et al. (1993) demonstrated that the critical amino acid in the MCA-13 epitope was at position 124, with this residue being phenylalanine in the MCA-13 reactive severe CTV strains, but tyrosine in the non-reactive mild strains. This antibody has been used extensively to test registered budwood source trees in the Florida Budwood Registration Bureau since it became mandatory in 1997 (Rucks, 1994; Kesinger, 2003). Trees in Florida which tested positive with MCA-13 could not be used as a source of budwood. In the Central California Tristeza Eradication Agency, the MCA-13 antibody is now used as a pre-screen test to flag CTV infected trees for further molecular and biological testing (Yokomi et al., 2012).

Analyses of the coat protein (CP) gene sequences of several CTV isolates having different biological activities led to the discovery that often CTV strains having similar biological activities show group-specific nucleotides at certain positions of the CP gene. This resulted in the development of the strain group specific probes (SGSP; Cevik, 1995; Niblett et al., 2000). Eight hybridization probes were designed: Probe 0 contains a sequence conserved in the CP gene of all known CTV isolates, and it serves as a universal probe to detect all CTV strains. Probe I hybridizes with CTV strains expressing decline and seedling yellows (T36, T66 are type isolates). Probe II hybridizes with CTV strains expressing decline, seedling yellows, and stem pitting on grapefruit and sweet orange (B1, B53 are type isolates). Probe III hybridizes with CTV strains expressing decline, seedling yellows, and stem pitting on grapefruit and sweet orange (B165, B185 are type isolates). Probe IV hybridizes with CTV strains expressing decline on sour orange, seedling yellow, and stem pitting on sweet orange (T3, B220 are type isolates). Probe V hybridizes with CTV strains expressing decline on sour orange, seedling yellows, stem pitting on grapefruit and sweet orange (B128, B249 are type isolates). Probe VI hybridizes with CTV strains which are very mild, such as found in Florida (T26, T30 are type isolates). Probe VII hybridizes with CTV strains which are mild, but commonly found in the Orient (B188, B215 are type isolates). Probe VIII hybridizes with all CTV strains that are mild, regardless of origin (T26, T30, B188, and B215 are the type isolates). The SGSP analyses have been useful in field surveys (Halbert et al., 2004), and in evaluation of CTV isolates being considered for mild strain cross protection (Ochoa et al., 2000). More information on the CTV isolates beginning with B (for Beltsville collection) may be found in Garnsey et al. (2005) and their biological activity as determined by biological indexing is summarized in **Table 1**.

**Table 1:** Summary of the biological activities of the type strains of CTV used in the strain group specific probe assays and other isolates of CTV referred to.

Isolate	Mexican lime	Sour orange	Grapefruit	Sweet orange	Sweet orange on sour orange
T36	2.0	2.0	1.0	0.0	1.5
T66	1.0	1.0	0.5	0.0	1.0
B1	3.0	3.0	2.0	0.0	3.0
B53	2.5	2.5	2.8	1.2	2.5
B165	2.5	2.2	3.0	2.5	2.5
B185	2.5	2.0	3.0	1.3	2.5
T3	2.5	2.0	1.0	0.5	2.0
B220	2.0	2.5	2.2	0.0	2.5
B128	2.5	0.5	2.2	0.5	1.0
B249	2.0	3.0	2.5	1.0	3.0
T26	0.5	0.0	0.0	0.0	0.0
T30	0.5	0.0	0.0	0.0	0.0
B188	1.2	0.0	0.0	0.0	0.0
B215	0.8	0.0	0.0	0.0	0.0
T3800	3.0	2.5	3.0	0.5	3.0
T55	0.5	0.0	0.0	0.0	0.0
T56	0.5	0.0	0.0	0.0	0.0
T60	1.0	0.0	0.0	0.0	0.0
T68	2.5	2.0	1.0	0.0	2.0

*Biological activities were determined as reported by Garnsey etal. (1987), 2005 using five different indicator plants, Mexican lime, sour orange, Duncan grapefruit, Madam vinous sweet orange, and sweet orange grafted onto sour orange rootstock. The plants are rated using a scale where 0 is healthy and 3 is most severe. The Mexican lime was rated for chlorosis, stunting, and stem pitting; sour orange for seedling yellows symptoms; Duncan grapefruit for seedling yellow, stem pitting, and stunting; Madam vinous sweet orange for chlorosis, stunting, and stem pitting, and the sweet orange on sour orange for decline.*

The single-stranded conformation polymorphism (SSCP) method is a useful approach to detect single base mutations in genes. This method has been applied to the CP gene of CTV (Rubio et al., 1996) and to the p18, p20, and p27 genes of CTV (Febres, 1995; Ayllón et al., 1999; Rubio et al., 2001). The amplified RT-PCR products are denatured and then electrophoresed on non-denaturing polyacrylamide gels. The denatured DNA strands form intra-molecular hydrogen bonds when entering the non-denaturing gel instead of annealing to their complementary strands, and are separated based on their relative conformations. After silver staining, there are usually two bands that can be visualized; multiple strains of CTV in the plant being tested produce a more complex band pattern. This often is used as a screen to select clones for sequencing of the samples.

## THE FLORIDA PROTOCOL FOR TIMELY SELECTION OF MILD STRAINS OF CTV FOR CROSS PROTECTION

When the BrCA arrived in Florida in 1995 and it was realized that the spread of CTV, including severe strains, was increasing (Halbert et al., 2004), we began efforts to select potentially useful mild strains for future use for mild strain cross protection. We will summarize this protocol as it was helpful to select promising mild isolates more rapidly than the empirical approach used previously.

**FIGURE 2:**
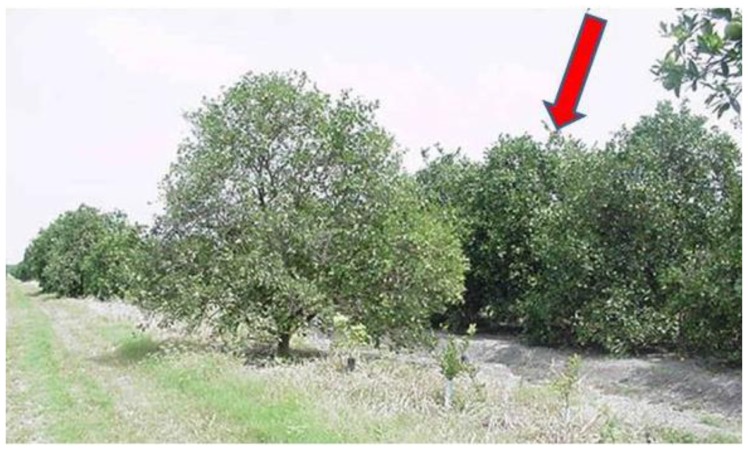
**An example of a surviving tree (red arrow) in a grove of Valencia sweet orange on sour orange rootstock.** Missing trees result in empty tree spaces or recently planted new trees. The trees surrounding the surviving tree show decline, general chlorosis in the tree canopy, and thinning of the canopy. The tree shown here was the source of one of the mild isolates evaluated under greenhouse conditions in Dekkers and Lee (2002).

The starting point was to pick isolates from groves being decimated with CTV, and in our instance the decimation was occurring in trees on sour orange rootstock. Budwood was collected from the best looking surviving trees in the field (see example in **Figure 2**). When the budwood was taken to the laboratory, the first test was to conduct MCA-13 ELISA and broad spectrum detection ELISA from each piece of budwood collected. The broad spectrum ELISA confirms the presence of CTV, and we were looking for mild isolates that are non-reactive in the MCA-13 ELISA.

**FIGURE 3:**
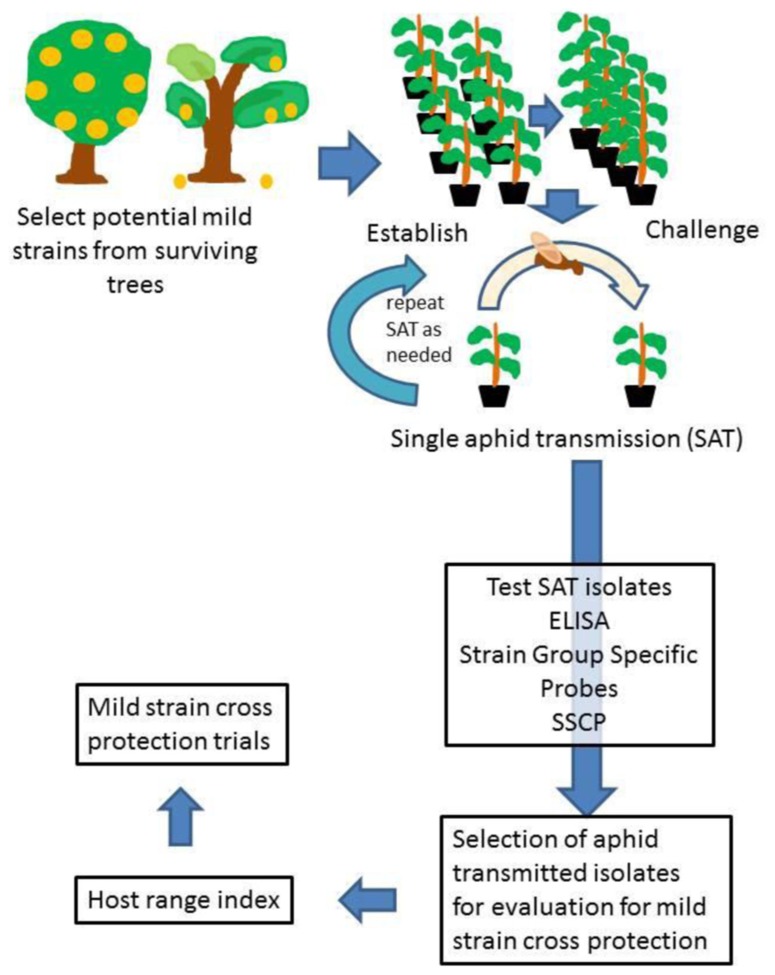
**Diagram showing the scheme followed to select mild isolates of CTV for use for mild strain cross protection**.

The budwood from surviving trees which had low reactivity in MCA-13 ELISA as compared to the broad spectrum detection ELISA were then propagated into eight sweet orange budlings propagated on sour orange rootstock using blind buds (no bud eyes; **Figure 3**). These budlings were held in small pots to minimize space required. Once the success of CTV graft transmission was confirmed by use of broad spectrum ELISA (usually 3 months), half of the budlings from each budwood source were challenged by graft inoculation of four severe CTV isolates (T36, T68, T66, and T3800) into each of the four plants. The bud take was monitored at two weeks after graft challenge, and plants reinoculated if buds had died. The plants were then held for 3–6 months, and the growth of the challenged plants compared to the growth of the four budlings which had only the CTV isolate recovered from the field. If the challenged plants continued to grow well, the four unchallenged budlings were retained, and the challenged plants discarded. If the challenged plants did not grow, or exhibited yellowing, all of the plants were discarded, the unchallenged plants and the challenged plants. This is a severe early test to select for potentially useful cross protecting CTV isolates, and for each isolate retained for further clean up and evaluation, probably 100 were discarded.

The next step was to do single aphid transmissions from the sources which had been selected. From a colony of BrCA maintained on healthy citrus, the aphids were transferred to tender young tissue on the source plants and allowed to remain for 24 h, after which the aphids were transferred to the young receptor plants, usually Madam vinous sweet orange seedlings, with one aphid per plant. After 24 h, the aphids were removed, the plants sprayed with an insecticide, and then placed back into the greenhouse. After 12–15 weeks, the receptor plants were checked by MCA-13 and broad spectrum detection ELISA to see if they were infected with CTV and if severe isolates were present. Most of the times the plants testing positive after the first round of single aphid transmission were subjected to another round of single aphid transmission as before.

The single aphid transmitted isolates were then subjected to molecular testing, using SGCP and SSCP of the p27 as well as retesting by MCA-13 and broad spectrum detection ELISA (Ochoa et al., 2000). Selection of isolates for host range testing was then made from isolates which appeared to consist of a single strain and not a mixture of strains based on either SGSP and SSCP analyses. 

The host range testing was performed on five indicator plants: Mexican lime, sour orange, Duncan grapefruit, Madam vinous sweet orange, and Hamlin sweet orange grafted onto sour orange using the protocol described by Garnsey et al. (1987; 1991; 2005). The results of indexing on all of the hosts except sweet orange on sour orange can be completed in 6–8 months. The biological host range test is important to make sure the selected mild strains are, in fact, mild.

The selected isolates are then ready for greenhouse testing and evaluation (Dekkers and Lee, 2002), after which the most promising isolates would go out to field trials. The selection of mild strains should be a continuous process as the dynamics of the CTV strains and populations in the field will be constantly changing, especially in areas where the BrCA has become established.

## USE OF MILD STRAINS OF CTV IN FLORIDA TO PROTECT AGAINST SEVERE CTV STRAINS

The decision of when to implement mild strain cross protection as a management strategy to limit CTV losses normally is made after the severe strains of CTV have become endemic and are causing economic losses, and there is little risk in widespread dissemination of mild strains. CTV decline on sour orange rootstock may be managed effectively by growing trees on a CTV-tolerant rootstock, however sour orange is a desirable rootstock because of the high fruit quality that it induces on grafted varieties. The Indian River production area in Florida is known for the high quality fruit, and most of the fresh fruit originates in this area. This quality is due in part to the use of sour orange rootstock. It was because of the demand for high quality fruit grown on sour orange rootstock that we began research to empirically select mild isolates of CTV which would protect against CTV decline on sour orange rootstock. In Florida, it was not until 2002 that the occurrence of a stem pitting isolate of CTV was reported in commercial citrus and shown to be spreading (Sieburth and Nolan, 2005). Most of the evaluation of Florida mild isolates for protection against CTV stem pitting strains has been done with foreign cooperators (van Vuuren et al., 1991; Ochoa et al., 1993; Vegas et al., 1995).

Several mild CTV isolates (T26, T30, and T55; **Table 1**) that are useful for mild strain cross protection against CTV-D have been selected empirically in Florida. Two different approaches have been used to protect against CTV-D strains which became common in Florida in the 1970s and 1980s. The first approach is to introduce the mild strain into budlings in the nursery, either by blind bud inoculation or by the use of budwood sources already infected with the desired mild strains. The performance of these cross protected budlings has been monitored by greenhouse trials and by field trials (Yokomi et al., 1987; Rocha-Pena and Lee, 1991; Rocha-Pena et al., 1991, 1992; Ochoa et al., 2000; Dekkers and Lee, 2002). The second approach is to introduce the mild CTV strain into mature (7–25 years old) trees on sour orange rootstock using blind buds or leaf piece inoculations, even though the trees are already infected with common and/or CTV-D strains (Lee and Brlansky, 1990; Lee et al., 1995; Lee, 2009). This approach was used in field situations where up to 20% tree loss was occurring annually. While trees on sour orange continue to decline, the rate of decline is slowed so that the production stays at a more consistent level, rather than having all the trees decline at once, then waiting 3 years before the newly planted trees on a CTV tolerant rootstock start to come into production.

Most of the data obtained on the effectiveness of mild strain cross protection has come from experiments which incorporate the mild strain into the budlings at the nursery level. More information has been obtained on the use of Florida mild strains to cross protect against stem pitting strains of CTV than their long term ability to cross protect against CTV-D strains. This is due to severe freezes in Florida in December 1983, January 1985, and December 1989 which destroyed most field plot experiments prior to their completion. Greenhouse evaluations have been utilized to obtain preliminary evaluation of the effectiveness of mild strain cross protection (Rocha-Pena et al., 1991, 1992; Dekkers and Lee, 2002).

Data obtained from cross protection experiments established in Florida suggests that cross protection is possible against CTV-D strains. One experiment was established in the DPI Foundation Grove, Dundee, FL, USA in 1985, exposed only to the natural challenge in that location. Before it was killed in the December 1989 freeze, blocks inoculated with three mild isolates (T30, T49, and T50a) had no declining trees, blocks inoculated with three other mild isolates (T55a, T56, and T60a) had only 10% decline, while those planted virus-free had 50% decline (Yokomi et al., 1992). Companion experiments were established at the Citrus Research and Education Center, Lake Alfred, FL, USA; one plot was challenged with the CTV-D isolate T36 using aphids, and in 1986 the second plot was graft challenged with CTV-D isolates T36 and T66. Testing with ELISA using the MCA-13 monoclonal antibody that reacts specifically with severe CTV isolates indicated severe strains were present in the trees, but the trees continued to grow well except for the occurrence of stunting in some trees (Rocha-Pena et al., 1991; Lee and Niblett, 2000) up until the freeze of 1989.

The inoculation of mild strains for cross protection into mature trees was studied beginning in 1987 (Lee and Brlansky, 1990). Earlier studies had indicated that some mild strains of CTV were able to spread throughout a tree canopy when inoculated into mature trees (Lee et al., 1988). Preliminary trials indicated that if branches at the four compass points on the canopy were inoculated, the T30 isolate of CTV, which is easily identified by a unique double stranded RNA pattern, was distributed throughout the tree canopy within 6 months (Lee et al., 1988, 1992). The first field trial was in a 12 year old grove of pineapple sweet orange on sour orange rootstock in the flatwoods production area where 20% of the trees were being killed annually due to CTV-D (Lee and Brlansky, 1990; Lee et al., 1992). There were seven single tree replications of mild strain T30, mild strain T26, and no mild strain inoculated trees. The inoculations were performed on the compass points of the tree using leaf piece inoculum. At the end of one year, the trees were rated on a 1–4 scale where 1 was healthy and 4 was dead, and the average value is for the trees still living. The control treatment (no mild strain) was 3.3 with 2 trees dead; the T30 treatment was 2.3 with 1 tree dead, and the T26 treatment was 1.8 with no trees dead. In 1987, a block of Navel sweet orange on sour orange rootstock was selected in the flatwoods area near Avon Park, FL, USA. Mild isolates T26, T30, T55, and T11 were inoculated into the 7 year old trees where CTV was causing the demise of 5% of the trees per year, using 5 by 5 tree blocks and four replications per treatment. By monitoring selected trees by double stranded RNA analyses, it became apparent that the non-inoculated trees had acquired mild strain T30 within the 1 year. When this was realized, in 1988, we selected a neighboring block in the same grove to use as a control block, separated by two roads and an irrigation canal; this control block of 500 trees had 2% missing trees due to CTV-D in 1989. In 1999, 11 years after the mild isolates had been introduced into the treatment plot, 89% of the original trees on sour orange rootstock still remained while in the control block, where mild strains were not introduced, had only 21% of the original trees on sour orange rootstock remaining (Lee, 2009). In 1993, trees in the FBRB Foundation Planting at Immokalee, FL, USA were inoculated with mild isolates T11, T26, T30, and T55 (Kesinger, 2003). Over 1 million budeyes were cut from this foundation block from 1989 to 1998 and used for propagations and budwood increase blocks by commercial citrus nurseries in Florida. While the performance of these budeyes/propagations were not monitored for performance in protecting against severe CTV isolates, this management practice did distribute a lot of budlings into the Florida citrus industry which were infected with mild CTV isolates. From inoculum provided by the University of Florida Citrus Research and Education Center, Lake Alfred, FL, USA to growers in Northern Lake, Orange, and Marion Counties from 1999 to 2003, an estimated 13,000 ha of existing citrus on sour orange rootstock were inoculated with mild CTV isolates (Lee, 2009).

The Florida mild isolates have been exported to Brazil, South Africa, and Venezuela as freeze dried infectious preparations (Garnsey et al., 1981). The freeze dried preparations were slash inoculated into receptor hosts (Müller et al., 1990). In each country, the CTV cultures were established *in planta* and then graft inoculated into a wide host range of citrus commonly grown in that country as well as hosts commonly used for biological indexing of CTV (Garnsey et al., 1987). Once all interested parties were satisfied that the introduced mild CTV isolates were in fact mild, cross protection evaluations were established under quarantine conditions, first in a greenhouse or screenhouse for a preliminary evaluation, then in a small scale field plot in an isolated location. The field plots were established on sour orange, a rootstock that normally would not allow trees surviving more than a few months because of the presence of severe CTV isolates in those countries. In South Africa, Florida mild strains T26, T55, T32, T33, T54, T30, and an Israel isolate, Micveh T, provided the best protection in Valencia on sour orange rootstock, both in tree growth and yield (van Vuuren et al., 1991). The same Florida mild isolates performed well on Mexican lime and grapefruit in other evaluation trials (van Vuuren et al., 1991). In Brazil, an experiment was established with Marsh grapefruit, Galego lime, Ponkan mandarin, and Pera sweet orange scions, all on sour orange rootstock, with other plants of the same scions on Rangpur lime as a CTV tolerant rootstock for comparison (Vegas et al., 1995). Nine Florida mild isolates, T11a, T26, T30, T30-132, 37-T4b, 49-T59, 50-T4, 53-T35b, and 58-T37, along with two Brazil isolates, no. 50 and SP-Brazil Satsuma, were inoculated to six trees of each scion in a replicated block. All plants tested positive by MCA-13 ELISA indicating the presence of severe CTV strains. After one year in the field on sour orange rootstocks, Florida isolates 30-T4, T11a, and T30 provided the best growth on Pera sweet orange; T11a and T30a provided the best growth on Galego lime; isolates T26, and 53-T35b provided the best growth on Marsh grapefruit along with a Brazilian isolate no. 50. With the Ponkan mandarin scions, the two Brazil isolates, no. 50 and SP-Brazil Satsuma, provided the best growth. At the end of 3 years in the field, the Pera, Galego lime, and Ponkan mandarin scions were all unthrifty, the Marsh grapefruit scions on sour orange preimmunized with no. 50 CTV isolate from Brazil were still growing and producing, but much smaller than the same scion on Rangpur lime rootstock. In another trial in Brazil, Ponkan mandarin, Marsh grapefruit, Galego lime, Pera, Folha Murcha, and Hamlin sweet oranges were propagated on GouTouCheng sour orange hybrid rootstock and preimmunized with Florida mild isolates T26 and T30 and Brazil mild isolate no. 50. After 9 years, all the preimunized trees were still growing satisfactorily with little stem pitting and bearing good crops. The authors concluded that the Florida mild isolates may provide good protection against CTV-SP strains in the presence of the BrCA if the trees are on a CTV-tolerant rootstock (Vegas et al., 1995). In Venezuela, a field trial was established to evaluate the performance of Valencia sweet orange on sour orange rootstock preimmunized with three Florida mild isolates, T26, T30, and T30a, and eight Venezuela isolates of CTV. After 3 years of evaluations, trees preimmunized with Florida T30 mild isolate continued to grow satisfactorily whereas the other trees were stunted and showed vein corking and stem pitting (Ochoa et al., 1993).

## WHAT DOES THE FUTURE HOLD FOR MILD STRAIN CROSS PROTECTION OF CTV?

*Citrus tristeza virus *with a single stranded RNA genome of ~19.3 kb presented a challenge in construction of an infectious clone, but in 1999 the infectious clone of CTV isolate T36 was reported by Satyanarayana et al. (1999) Since that time, with the use of the infectious clone of T36, much has been learned about the expression strategies of CTV, genetic variability, and the infectious clone with a green fluorescent protein (GFP) label has been useful for evaluating transgenic plants for resistance to CTV (Dawson, 2010). As the molecular biology of CTV has been studied, better and more sensitive diagnostic procedures have been developed and applied for studies on cross protection and epidemiology. The resistance in *P. trifoliata* has been identified and characterized, and this may be useful in developing resistance at least to most of the isolates of CTV in the future (Mirkov et al., 2010). However, *P. trifoliata* resistance breaking isolates of CTV have been reported, first in New Zealand and later in other locations (Dawson and Mooney, 2000). Transgenic resistance has been reported in grapefruit, but this has not been used on a commercial scale (Febres et al., 2008). More recently, transformation of Mexican lime with an intron-hairpin construct expressing untranslatable versions of the genes coding for the three silencing suppressors of CTV (Lu et al., 2004) conferred complete resistance to the same genotype of CTV (Soler et al., 2012). There are many things still to be learned about CTV; where are the pathogenicity factors located and what interactions do they have with the host to impart resistance or tolerance? We now know that defective RNAs commonly occur with CTV, but it is still to be discovered what role they play in the biology and replication of CTV in various hosts. We now know that CTV has three potentially gene silencing suppressors (Dawson, 2010); as the regulation of these genes becomes better understood, they may be useful for protecting against severe isolates of CTV in commercial crops.

It was recently reported that infection with one strain of CTV excludes infection by another isolate of the same strain of CTV (Folimonova et al., 2010). Using CTV isolates generated from the infectious clone, the inoculation first with T36 strain prevented subsequent infection when the same plants were inoculated with T36 labeled with GFP, but when other CTV strains were used, there was no apparent effect on replication or movement of the challenge virus. This discovery may be useful in the future where possibly the severe CTV isolate could be genetically characterized and a mild variant created using an infectious clone. The mild variant could then be used as the protecting isolate, providing protection against later infection by the severe isolate. There will need to be some breakthrough in technology before this becomes a reality. We know now that even well characterized isolates of CTV may contain “hidden” severe strains that may become apparent later, usually by aphid transmission (Albiach-Marti et al., 2000; Tsai et al., 2000; Brlansky et al., 2003). While the cost of fully sequencing the genome of a CTV isolate has become very reasonable, the resulting sequence is often a consensus of the population present in the sample, and probably would not detect the presence of minor, but potentially severe, strains of CTV in the isolate. Also, it is not easy to construct an infectious clone, and the T36 infectious clone is the only one reported to date. While many of the 3′ end genes have been substituted for those of other CTV isolates, the report by Folimonova et al. (2010) suggests they will not work to prevent superinfections.

In the future, technology will be developed which will allow identification of severe strains of CTV in a given isolate, and with a much better understanding of how superinfections can be prevented, cross protection may be applied using a much more intelligent approach with molecular tools. For the foreseeable future, the empiric approach, coupled with improved diagnostic ability to quickly and accurately detect and differentiate among CTV strains, will still be the most productive approach for developing mild strain cross protection against CTV as additional citrus production areas experience the introduction and spread of severe CTV which limits production.

## Conflict of Interest Statement

The authors declare that the research was conducted in the absence of any commercial or financial relationships that could be construed as a potential conflict of interest.
